# Transcutaneous penetration of a single-chain variable fragment (scFv) compared to a full-size antibody: potential tool for atopic dermatitis (AD) treatment

**DOI:** 10.1186/s13223-021-00574-x

**Published:** 2021-07-19

**Authors:** Audrey Baylet, Raoul Vyumvuhore, Marine Laclaverie, Laëtitia Marchand, Carine Mainzer, Sylvie Bordes, Brigitte Closs-Gonthier, Laurent Delpy

**Affiliations:** 1 Unité Mixte de Recherche CNRS, 7276-INSERM U1262-Université de Limoges, CBRS, 2 rue du Dr Marcland, 87025 Limoges, France; 2 Silab R&D Department, Brive, France

**Keywords:** Transcutaneous penetration, Single-chain variable fragment, Antibody, Atopic dermatitis

## Abstract

Currently, several biologics are used for the treatment of cutaneous pathologies such as atopic dermatitis (AD), psoriasis or skin cancers. The main administration routes are subcutaneous and intravenous injections. However, little is known about antibody penetration through the skin. The aim was to study the transcutaneous penetration of a reduced-size antibody as a single-chain variable fragment (scFv) compared to a whole antibody (Ab) and to determine its capacity to neutralize an inflammatory cytokine involved in AD such as human interleukin-4 (hIL-4). Transcutaneous penetration was evaluated by ex vivo studies on tape-stripped pig ear skin. ScFv and Ab visualization through the skin was measured by Raman microspectroscopy. In addition, hIL-4 neutralization was studied in vitro using HEK-Blue™ IL-4/IL-13 cells and normal human keratinocytes (NHKs). After 24 h of application, analysis by Raman microspectroscopy showed that scFv penetrated into the upper dermis while Ab remained on the *stratum corneum*. In addition, the anti-hIL4 scFv showed very efficient and dose-dependent hIL-4 neutralization. Thus, scFv penetrates through to the upper papillary dermis while Ab mostly remains on the surface, the anti-hIL4 scFv also neutralizes its target effectively suggesting its potential use as topical therapy for AD.

**Supplementary Information:**

The online version contains supplementary material available at 10.1186/s13223-021-00574-x.

## Introduction

Currently, immunotherapy is used for the treatment of several skin pathologies such as atopic dermatitis (AD), psoriasis (PSO) and cancers, mainly via subcutaneous or intravenous routes. Finding new antibody delivery methods is a challenge and different technologies have emerged in recent years such as microneedle patches, nanoparticles, liposomes or gel formulations [1]. In addition, several modified antibodies are under development to treat cutaneous diseases, but at present, only certolizumab pegol, a PEGylated anti-tumor necrosis factor α antigen-binding fragment (Fab) has been approved (2013) for the treatment of PSO [1]. AD is an inflammatory skin disease with a high and constantly increasing prevalence worldwide (1–3% of adults and 15–20% of children) [2]. This pathology is characterized by a skin barrier default and a Th2 phenotype with secretion of several proinflammatory cytokines such as interleukin-4 (IL-4), IL-5 and IL-13. Among them, IL-4 is particularly involved in immunoglobulin (Ig) class switching to IgE [3]. Different cell populations express the high affinity IgE receptor (FcεRI) and immune complex phagocytosis by these cells leads to T lymphocytes activation [4]. Until now, only one immunotherapy treatment targeting the IL-4/IL-13 common receptor, IL-4 receptor alpha (IL-4Rα), has been approved for AD. Thus, developing a new topical immunotherapy treatment is a real challenge that would improve patients quality of life.

At present, there is no evidence in the literature proving that a reduced-size antibody could penetrate the skin more easily than a whole antibody. Thus, the first aim was to compare the passage of a single chain variable fragment (scFv) (~ 25 kDa) through a damaged skin, mimicking the poor barrier function of atopic skin, compared to a full-size monoclonal antibody (Ab) (~ 150 kDa). In addition, we addressed whether a scFv could neutralize the effects of human IL-4 (hIL-4) after stimulation of normal human keratinocytes (NHKs).

## Methods

For the comparative study of the transcutaneous penetration of whole antibody and scFv, ex vivo experiments were performed on pig ear skin*.* After shaving, ears were tape-stripped 25 times (Clinical&Derm, USA) to obtain the altered skin condition. Then, 8 mm diameter punches were treated with 5 µl phosphate buffered saline (PBS, Corning, USA), and 11 µM anti-CD44 scFv (Creative Biolabs, USA) or anti-CD44 Ab (Thermofisher Scientific, USA). Skin samples were placed at 37 °C for 6 h or 24 h and cryopreserved until Raman microspectroscopy analysis.

Three 14 µm thick sections were used per sample and three Raman acquisitions were read for each section totaling nine measures per sample. Briefly, Raman images were obtained using a confocal Raman microspectrometer (Horiba Jobin Yvon, France) operating with a 660 nm laser. Labspec 6 software (Horiba Jobin Yvon, France) was used for acquisition and data pre-processing. Raman maps were recorded in an area of X: 10-µm/Y: 150-µm with 5 µm step size in the XY directions. Raman spectra were acquired in the 400–2300 cm^−1^ spectral range. To prevent background noise, Raman spectra were smoothed using a 2nd order Savitzky-Golay type filter, baseline corrected using a 7th order polynomial function, and spectra with an intensity under 400 cts in the 1530–1730 cm^−1^ range were suppressed. Next, Raman spectra were cropped to keep only the 400–785 cm^−1^ range of interest, which was used for vector normalization. Finally, fitting (unmixing) by classical least squares with a non-negativity constraint (NCLS) was used to estimate the contribution of the various skin components leading to the images reflecting the distribution of the specific scFv or Ab through the skin cryo-sections.

To assess the neutralizing capacity of anti-hIL4 scFv in vitro, we used both a cell line model (HEK-Blue™ IL-4/IL-13 cells; InvivoGen, France) and NHKs as primary cells. HEK-Blue™ IL-4/IL-13 cells were cultured according to manufacturer’s instructions. Briefly, 50,000 cells were seeded in 96-well plates. Then, cells were stimulated with 10 ng/ml hIL-4 (Miltenyi Biotec, Germany) and treated with anti-hIL4 scFv (Creative Biolabs, USA) or Ab (Biotechne, USA) at different concentrations. The variable domains of the anti-hIL4 scFv and monoclonal Ab are distincts and the latter was used as a positive control. After 24 h, QUANTI-Blue™ substrate was added to supernatants. After 2h30 at 37 °C, SEAP levels were measured at 620 nm using a spectrophotometer.

NHKs were isolated from human skin samples from healthy donors (*n* = 4) undergoing medical surgery. The skin was collected after written informed consent from the donors and institutional approval. NHKs were stimulated with polyinosinic-polycytidylic acid (poly I:C) (2 µg/ml, Sigma, USA) ± hIL-4 (25 ng/ml, Peprotech, USA) and treated with anti-hIL4 scFv at different concentrations. After 24 h, supernatants were harvested to evaluate hIL-8 secretion by enzyme-linked immunosorbent assay (ELISA) according to manufacturer’s instructions (R&D Systems, USA). Cell protein concentrations were determined using the BCA Protein Assay kit (Thermofisher Scientific, USA) according to manufacturer’s instructions. hIL-8 secretion was normalized to total protein amounts.

Statistical analysis was performed using Prism GraphPad software (GraphPad Software, USA). Significant differences between samples and control were evaluated by Student’s *t*-test. *P* values < 0.05 were considered significant.

Some detailed protocols are available in the Supporting Information.

## Results

**Fig. 1:**
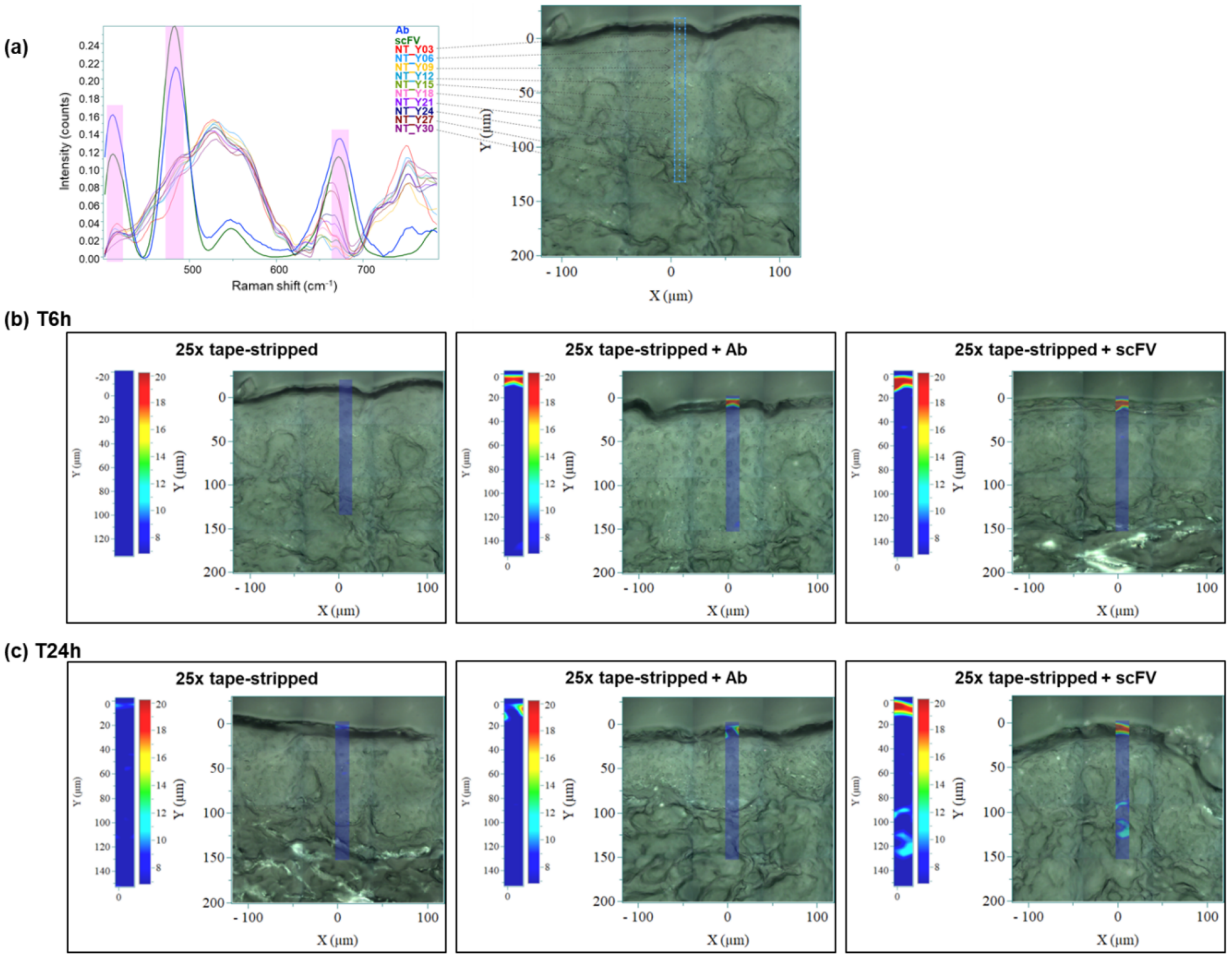
Visualization of transcutaneous antibody penetration by Raman microspectroscopy. **a** Single-chain variable fragment (scFv) and antibody (Ab) Raman signals (highlighted in pink) and skin Raman signals at different depths between 400 and 785 cm^−1^. **b** Quantification of scFv and Ab in damaged skin after 6 h or (**c**) 24 h of treatment

First, we used Raman microspectroscopy to assess the transcutaneous penetration of antibody formats using pig ear skin. Three distinct peaks from the skin signal, corresponding to the scFv and Ab specific Raman signatures, were identified in the spectral range between 400 and 785 cm^−1^ (Fig. 1a). This first analysis was necessary to visualize antibody penetration through the skin. At 6 h, both scFv and Ab were only observed in the *stratum corneum* (depth: 10–20 µm) (Fig. 1b). At 24 h, scFv penetrated to a depth of 130 µm, corresponding to the upper papillary dermis while Ab remained on the surface (Fig. 1c). Results were obtained on altered skin samples. Hematoxylin and eosin staining (H&E) of pig ear skin showed an altered skin barrier function on tape-stripped samples due to removal of part of the *stratum corneum* (Additional file 1: Figure S1).

**Fig. 2:**
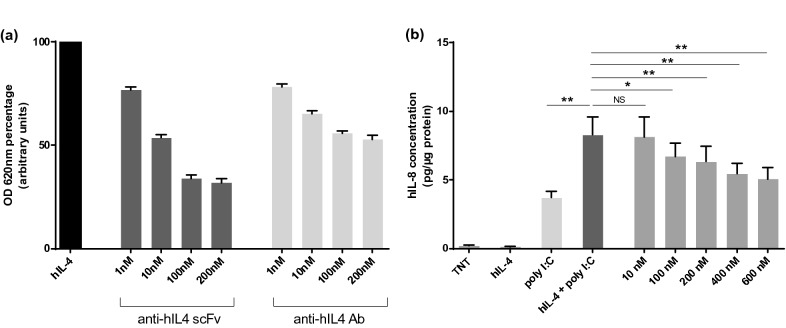
Human interleukin 4 (hIL-4) neutralization by single-chain variable fragment (scFv) on 2D models. **a** hIL-4 neutralization by scFv and Ab on HEK-Blue™ IL-4/IL-13 cells. Optical densities at 620 nm (OD 620 nm) were determined and results were expressed in arbitrary units after normalization to the maximum value (100%) measured in cells stimulated with 10 ng/ml hIL-4. In each experiment and for each condition, cells were seeded in triplicates (*n* = 4 for 1, 10 and 10 nM doses; *n* = 3 for 200 nM dose). Results are expressed as mean ± SEM. **b** Human interleukin 8 (hIL-8) quantification in supernatants of normal human keratinocytes (NHKs) stimulated with hIL-4 ± polyinosinic-polycytidylic acid (poly I:C) (25 ng/ml and 2 µg/ml, respectively) and treated with anti-hIL4 scFv at different concentrations for 24 h (*n* = 4). For the four donors, experiments were performed in duplicates. Results are normalized in relation to the quantity of total proteins per well and expressed as mean ± SEM (*NS* non-significant; *P < 0.05; **P < 0.01; ***P < 0.001; ****P < 0.0001)

Next, in vitro experiments were performed to assess the neutralizing capacity of anti-hIL4 scFv in HEK-Blue™ IL-4/IL-13 cells. Both the anti-hIL4 scFv and Ab showed significant dose-dependent neutralization of hIL-4 in the HEK Blue 2D model (Fig. 2a). In addition, at the maximal concentration of 200 nM, scFv neutralized an average of 68% of hIL-4 (Additional file 1: Table S1).

NHKs were then stimulated to evaluate hIL-4 neutralization by scFv on primary cells. hIL-8 dosage of supernatants from NHKs stimulated with hIL-4 ± poly I:C and treated with anti-hIL4 scFv, revealed a scFv dose-dependent decrease in four different donors (Fig. 2b). At 100, 200, 400 and 600 nM doses, a significant decrease in hIL-8 secretion was observed. ScFv neutralization ability against hIL-4 was calculated based on poly I:C induction of hIL-8. The hIL-4 + poly I:C was considered as the 0% of hIL-4 neutralization value, whereas poly I:C alone was considered as the 100% value (Table 1). At 600 nM scFv, the average percentage of hIL-4 neutralization reached 80%. Of note, the anti-hIL4 Ab used in this study showed low neutralization efficiency in this 2D model (data not shown).

**Table 1:** hIL-4 neutralization by scFv in normal human keratinocytes (expressed as a relative percentage to poly I:C)

Donors	poly I:C	hIL-4 + poly I:C	scFv 10 nM	scFv 100 nM	scFv 200 nM	scFv 400 nM	scFv 600 nM
D1	100	0	− 39	4	26	44	84
D2	100	0	69	84	118	113	127
D3	100	0	3	21	31	46	43
D4	100	0	− 7	33	28	58	64
Average (%)	**100**	**0**	**7**	**36**	**51**	**65**	**80**
Standard deviation (%)	0	0	45	35	45	32	36

*hIL-4* human interleukin 4, *poly I:C* polyinosinic-polycytidylic acid, *scFv* single chain variable fragment, *nM* nanomolar

## Discussion

Until now, few studies assessing antibody penetration in the skin have been carried out. Among them, topical application of infliximab, an anti-TNFα Ab, has been described in patients with ulcers [5], and Flightless I (Flii) neutralizing antibodies (FnAb) have been applied in a murine model of epidermolysis bullosa acquisita [6, 7]. No comparative study examining the penetration of reduced-size versus whole antibodies has yet been performed, especially using Raman microspectroscopy, which is a relevant tool for antibody characterization. Indeed, this technique can be used to monitor post-translational modifications, degradation or aggregation [8–10]. For several years, it has also been used to study the skin [11, 12]. To our knowledge, we are the first to track antibody passage through the skin using Raman microspectroscopy. Here, we showed in an ex vivo model of damaged skin that reduced size facilitated antibody penetration into the upper papillary dermis.

Only one antibody fragment has been approved for the treatment of skin pathologies. Recently, M1095, an anti-IL17A/F nanobody completed a phase 1 clinical trial for the treatment of moderate to severe PSO [13]. Thus, antibody fragments could be potential tools for the development of new topical treatments for cutaneous diseases. Indeed, in recent years, innovative immunotherapy methods have emerged including microneedles, nanoparticles or liposomes [14–17]. To date, only one immunotherapy treatment has been approved for AD. Several treatments targeting proinflammatory molecules including cytokines and their receptors are under development for AD management [18]. First, we chose to target hIL-4, which is a key cytokine involved in AD physiopathology. In fact, it leads to B-lymphocytes class switching to IgE and naïve T cells differentiation into Th2 [19]. Therefore, we decided to focus on the effect of hIL-4 neutralization on inflammatory human keratinocytes. We found a scFv dose-dependent decrease in hIL-8 secretion suggesting a key role for hIL-4 in inflammation. In addition, our results showed a strong neutralization efficiency of anti-hIL4 scFv on HEK-Blue™ IL-4/IL-13 cells.

A limitation of our ex vivo model could be the lack of lesional AD human skin biopsies. Tape strips represent a simple and easy way to mimic barrier disruption, feature observed in the pathology. Nevertheless, this alteration does not reflect all structural changes of atopic skin, suggesting a different behavior of scFv penetration on patient’s skin that would need to be assessed. In the future, it would be interesting to evaluate the benefits of a topical anti-mouse IL-4 treatment on an in vivo AD model such as NC/Nga mice [20].

To sum up, we showed that reduced-size antibodies depict better penetration abilities than full-size antibodies and retain high capacity to neutralize a cytokine target. Reduced-size antibodies could be therefore potential relevant topical treatments for inflammatory skin diseases as AD.

## Supplementary Information

**Additional file 1**: **Fig. S1**. Representative images for hematoxylin and eosin staining (H&E) of pig ear skin samples (a) with no treatment or (b) times tape-stripped to mimic damaged skin. **Table S1**: hIL-4 neutralization by scFv in HEK-Blue™ IL-4/IL-13 cells (expressed as a relative percentage to OD620 nm).

## Data Availability

The datasets used and/or analysed during the current study are available from the corresponding author on reasonable request.
