# Supplementary material for: Transcutaneous penetration of a single-chain variable fragment (scFv) compared to a full-size antibody: potential tool for atopic dermatitis (AD) treatment
Source: Allergy Asthma Clin Immunol. 2021 Jul 19;17:73. doi: 10.1186/s13223-021-00574-x (PMC8290589; doi:10.1186/s13223-021-00574-x)
Supplement: Supplementary file 1 — Additional file 1: Fig. S1. Representative images for hematoxylin and eosin staining (H&E) of pig ear skin samples (a) with no treatment or (b) times tape-stripped to mimic damaged skin. Table S1: hIL-4 neutralization by scFv in HEK-Blue™ IL-4/IL-13 cells (expressed as a relative percentage to OD620 nm). [file 13223_2021_574_MOESM1_ESM.pdf]

Journal of Allergy, Asthma and Clinical Immunology

Transcutaneous penetration of a single-chain variable fragment (scFv) compared to a full-size antibody: potential tool for Atopic Dermatitis (AD) treatment

Audrey Baylet[1,2], Raoul Vyumvuhore[2], Marine Laclaverie[2], Laëtitia Marchand[2], Carine Mainzer[2], Sylvie Bordes[2], Brigitte Closs-Gonthier[2,*], and Laurent Delpy[1,*,#]

Supplementary material

HEK-Blue™ IL-4/IL-13 cells. IL-4/IL-13 binding to their common receptor subunit, IL-4 receptor alpha (IL-4Rα), leads to signal transducer and activator of transcription 6 (STAT6) phosphorylation by Janus kinases (JAK) and its translocation in the nucleus to activate target genes. HEK-Blue™ IL-4/IL-13 cells (InvivoGen, France) specifically express the reporter gene secreted embryonic alkaline phosphatase (SEAP) under the control of a specific promotor fused to several STAT6 binding sites. After stimulation with hIL-4, SEAP was detected in culture supernatants using QUANTI-Blue™ substrate.

Normal human keratinocytes (NHKs). NHKs were cultured in Keratinocyte serum-free medium (KSFM) (Thermofisher Scientific, U.S.A.) supplemented with human recombinant epidermal growth factor (rEGF) and bovine pituitary extract (BPE) at the time of use and were harvested at third-passage by trypsin digestion. At day 0, 40 000 cells per well were seeded in 24-well plate in KSFM rEGF BPE. At 80% confluence, NHKs were stimulated with polyinosinic-polycytidylic acid (poly I:C) (2 µg/ml, Sigma, U.S.A.) +/- hIL-4 (25 ng/ml, Peprotech, U.S.A.) and treated with anti-hIL4 scFv (10, 100, 200, 400 and 600 nM) diluted in 500 µl KSFM rEGF BPE. For each condition, experiments were carried out in duplicates. After 24h, supernatants were harvested to evaluate hIL-8 secretion by enzyme-linked immunosorbent assay (ELISA) according to manufacturer's instructions (R&D Systems, U.S.A.). Cells were lysed by adding 300 µl of NaOH 0.1N and cellular proteins concentration

was determined using the BCA Protein Assay kit (Thermofisher Scientific, U.S.A.) according to manufacturer's instructions.

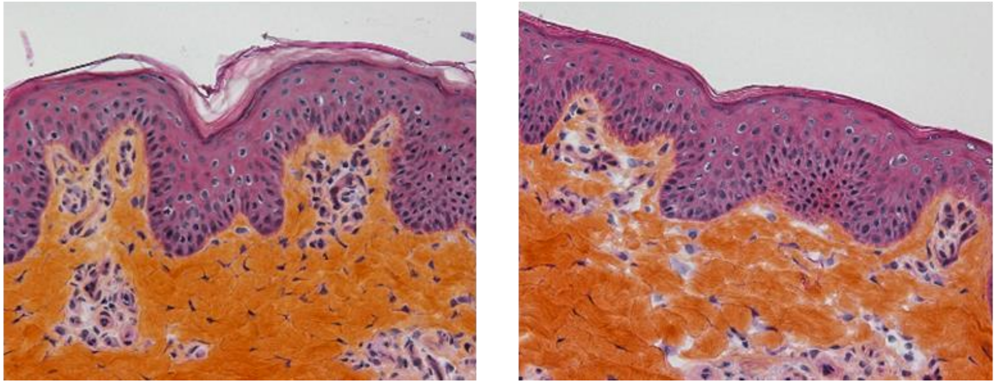

Supplementary Figure S1. Representative images for hematoxylin and eosin staining (H&E) of pig ear skin samples (a) with no treatment or (b) 25 times tape-stripped to mimic damaged skin.

Supplementary Table S1: hIL-4 neutralization by sccFv in HEK-Blue™ IL-4/IL-13 cells (expressed as a relative percentage to OD620nm)

	scFv (nM)				Mab (nM)				
	**1**	**10**	**100**	**200**	**1**	**10**	**100**	**200**	**No treatment**
**Exp. 1**	27	49	69		24	31	41		
**Exp. 2**	24	49	72	75	23	41	51	53	0
**Exp. 3**	18	41	61	64	24	32	44	44	0
**Exp. 4**	24	46	62	65	16	34	42	45	0

**Average (%)**	**23**	**46**	**66**	**68**	**22**	**35**	**44**	**47**	**0**
**Standard deviation (%)**	4	4	5	6	4	4	4	5	0

hIL-4: human interleukin 4. IL: interleukin. scFv: single chain variable fragment. Exp.: experiment. nM: nanomolar.
